# Clinical Studies on Treatment of Earthquake-Caused Posttraumatic Stress Disorder Using Electroacupuncture

**DOI:** 10.1155/2012/431279

**Published:** 2012-09-25

**Authors:** Yu Wang, You-ping Hu, Wen-chun Wang, Ri-zhao Pang, An-ren Zhang

**Affiliations:** ^1^Department of Rehabilitation, Chengdu Military General Hospital, No.270, Road Rongdu, Jinniu District, Chengdu 610083, China; ^2^Acupuncture and Tuina College, Chengdu University of Traditional Chinese Medicine, Chengdu 610075, China

## Abstract

The objective of this study was to assess the efficacy and safety of electroacupuncture in 138 patients with earthquake-caused PTSD using Randomized Controlled Trials (RCTs). 138 cases enrolled were randomly assigned to an electro-acupuncture group and a paroxetine group. The electro-acupuncture group was treated by scalp electro-acupuncture on Baihui (GV 20), Sishencong (EX-HN 1), Shenting (GV 24), and Fengchi (GB 20), and the paroxetine group was treated with simple oral administration of paroxetine. The efficacy and safety of the electro-acupuncture on treatment of 69 PTSD patients were evaluated using Clinician-Administered PTSD Scale (CAPS), Hamilton Depression Scale (HAMD), Hamilton Anxiety Scale (HAMA), and Treatment Emergent Symptom Scale (TESS) according to clinical data. The total scores of CAPS, HAMD, and HAMA in the two groups after treatment showed significant efficacy compared to those before treatment. The comparison of reduction in the scores of CAPS, HAMD, and HAMA between the two groups suggested that the efficacy in the treated group was better than that in the paroxetine group. The present study suggested that the electro-acupuncture and paroxetine groups have significant changes in test PTSD, but the electro-acupuncture 2 group was more significant.

## 1. Introduction

Posttraumatic stress disorder (PTSD) develops in some people after they have been exposed to a traumatic event such as sexual abuse, a serious road traffic collision, a natural disaster, criminal victimization, or military combat. PTSD patients exhibit depressive symptoms, abnormality of the circulating levels of the stress hormones and neurotransmitter activity, and alteration of gene expression [1]. Moreover, PTSD is also associated with blood pressure, bronchial asthma, peptic ulcer disease, obesity, cancer, and other psychosomatic diseases [2]. PTSD symptoms have been around for long time, but the significance of PTSD came to public attention only recently. PTSD is a serious public health concern, which compels the search for novel paradigms and theoretical models to deepen the understanding of the condition and to develop new and improved modes of treatment intervention. There are ongoing clinical controversies about the optimal diagnosis and treatment of PTSD, and the relevant research literature has burgeoned in recent decades [3, 4]. Due to the long-lasting and resistant symptoms, treatment of PTSD is complex and may involve the use of various treatment modalities, involving both pharmacotherapy and various types of psychotherapy, as reported in the majority of studies [5].

Based on the previous reports, the prevention and therapy methods of PTSD are mainly psychopharmacology, psychotherapeutic interventions, psychoeducation and support, and so forth. Psychopharmacology proved to be partially effective in reduction of symptoms of increased irritability and reexperiencing, but most often it had no effect on symptoms of avoidance. Along with new developments in neurophysiology of stress response antidepressants, anxiolytics, antiadrenergic agents, anticonvulsants, benzodiazepines, atypical antipsychotics, and novel agents came to be used for drug treatment, but the results were only partially satisfactory. Moreover, the limitations of conventional psychopharmacologic treatments were more expensive and were difficult to formal treatment for patients in low-income countries [1, 6]. Psychotherapeutic interventions mainly help to stabilize the mood and to restore the psychological balance of patients by cognitive behavioral therapy and professional psychological guidance. This method has positive impacts on the patient's symptoms, but the anxiety and vigilance of patients will affect the effects of treatment [3]. Supportive interventions are often used as the control intervention in studies of more specific treatments. However, clinical experience indicates that both support and psychoeducation appear to be helpful as early interventions to reduce the psychological sequelae of exposure to mass violence or disaster. When access to expert care is limited by environmental conditions or reduced availability of medical resources, rapid dissemination of educational materials may help many persons to deal effectively with subsyndromal manifestations of trauma exposure [7]. To date, there is no one best method specifically for the treatment of PTSD. Thus, an efficient, smaller side effect, and lower costs method for PTSD treatment will become the focus in clinical practice and research.

Natural disasters, like earthquakes, floods, fires, SARS, and so forth, not only cause losses of people's lives and property, but also result in people's psychological trauma. At 2:28 p.m. Beijing time, on May 12, 2008, an earthquake measuring 8.0 magnitude on the Richter scale, with its epicenter in Wenchuan county, hit a number of cities and counties in the southwest of China [8]. This earthquake put many people under unimaginable psychological pressure of horror, nightmare, helplessness, high alert, and other symptoms. Thus, PTSD is also the one consequence of earthquake trauma. Report from Zhao et al. suggested that the incidence rate of acute stress disorder (ASD) was 12.59% (110/874), and the incidence rates of ASD for female and male were 15.16% (72/475) and 9.52% (38/399), respectively [9]. Survey from Xiang-lan Wang et al. showed that the percentage of PTSD incidence, symptoms of upset when reminded, intrusive memory, and hypervigilance are 14.3%, 65.0%, 63.2%, and 51.6%, respectively [10]. Results from Fan et al.'s reports showed that 15.8%, 40.5%, and 24.5% of participants reported clinical symptoms of PTSD, anxiety, and depression, respectively [11]. These reports suggested that PTSD is one of the serious consequences of these traumatic events and will seriously affect the patient's mental health and life quality. Thus, the prevention and early diagnosis methods of PTSD have become among the important topics of basic medical research and clinical practice.

Acupuncture has been used for medicine with a continuous clinical history, such as emotional disorders, pain tissues, and postoperative nausea [12]. There is a larger literature of the positive effects of acupuncture for treating depression and insomnia [13–16]. Some reports also provide acupuncture protocols for depression, anxiety, and several other psychiatric disorders. Therefore, acupuncture protocols for PTSD are both logical and based on a long history of clinical use for psychiatric symptoms, which have also substantiated efficacy [13]. Electro-acupuncture, the integration of traditional Chinese acupuncture and modern electrotherapy, has been used for clinical treatment of analgesia and stroke [17, 18]. All these acupuncture systems are prevalent in current clinical practice, and the highly variable nature of acupuncture needling creates challenges to systematic research. However, there is yet a large sample and randomized controlled trial clinical research about electro-acupuncture head points on PTSD treatment. Thus, the aim of this study is to develop an electro-acupuncture treatment protocol for PTSD based on the Baihui (GV 20), Shenting (GV 24), Sishencong (EX-HN), and Fengchi (GB 20) points, and evaluate the potential efficacy and safety of acupuncture for treating PTSD in a randomized controlled trial (RCT). Information from this study will be helpful to provide a new way to prevention and treatment of PTSD. 

## 2. Method

### 2.1. General Data

512 Wenchuan earthquake-caused PTSD patients in the disaster areas were collected and admitted in Traditional Chinese Medicine Hospital of Mianyang City, Traditional Chinese Medicine Hospital of Jiangyou City, and Psychiatric Hospital of Dujiangyan City from May 2008 to November 2008. Participants were initially screened by telephone, and full assessments were conducted only for participants who did not report any exclusion criteria during the telephone screening. All participants provided written informed consent approved by the Hospital Human Research Ethics Committee.

### 2.2. Diagnosis and Inclusion Standards

Justification of the selected outcomes and interventions was for the most part clearly stated. In reference to the standard for diagnosing PTSD in the *Diagnostic and Statistical Manual of Mental Disorder*s, *Fourth Edition, Text Revision* (DSM-IV-TR) PTSD diagnostic criteria (p. 468), the subjects included were (1) those who 512 Wenchuan earthquake-caused masses, relief officers and volunteers; (2) those who their ages were between 18 and 65 years; (3) those who signed informed consent by himself or their immediate family members; (4) those who have clear consciousness, and no severe heart, liver, kidney disorders, and are able to participate in the examination and treatment.

### 2.3. Exclusive Standard

(1) Those who have suffered from depression or other mental disorders; (2) those who had the earthquake-caused severe damage with mentally retarded patients; (3) those who are taking anti-anxiety or antidepressant drugs; (4) those who are pregnant or lactating women.

### 2.4. Method of Treatment

**Figure 1:**
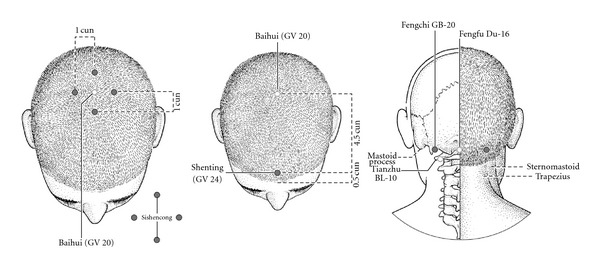
The locations of Baihui (GV 20), Sishencong (Ex-HN), Shenting (GV 24), and Fengchi (GB 20).

138 patients were randomized into an electroacupuncture group and a medication (paroxetine) group. There was no significant difference statistically in baseline characteristics of two groups. The medication (paroxetine) group received paroxetine (Zhejiang Huahai pharmaceutical Co., LTD, Linhai, Zhejiang, China) with simple oral administration 20 mg every night, and the treatment cycle is six weeks for two consecutive treatments (12 weeks). In the treatment group, the Sishencong (Ex-HN 1), Baihui (GV 20), Shenting (GV 24), and Fengchi (GB 20) points were selected by the introduction of Nomenclature and Location of Acupuncture Points (GB/T12346–006). Sishencong is a group of four points which are located on vertex of head, each 1 cun away from Baihui (GV 20) at four directions (anterior, bilateral, and posterior, Figure 1). Baihui is located on the head, 5 cun directly above the anterior hairline, and 7 cun directly above the posterior hairline. Shenting is located on the head, 0.5 cun directly above the midpoint of the anterior hairline. Fengchi is located in the neck, below the occipital bone, 1 cun above the posterior hairline. These acupuncture points are shown in Figure 1. These patients in the electro-acupuncture group were sitting by back against the wall, and the body was fixed. The skin of those acupoints was routinely disinfected using 75% ethanol. The disposable needle (*φ*  0.30 × 40 mm, Helio Medical Supplies, Inc., Suzhou, China) was obliquely needled into the point of galea capitis along the scalp up to 15~30° angle. The point of Fengchi was obliquely needled using 1.5 inch stainless steel needle, and the direction of needle tip was microdown on the tip of the nose about 0.5~1.2 inch. When the points of Shenting, anterior Shencong, and Baihui were needled, the direction of needles tip is forward. The directions of needle tip at left-sided, right-sided, and posterior Shencong are toward the Baihui point, and the deeps of needle are about 0.5~1 inch. These points were divided into two groups, and one group included the points of Shenting, Baihui, left-sided, and right-sided Shencong, and another group included the points of anterior Shencong, posterior Shencong, left-sided, and right-sided Fengchi. These needles in two groups were connected with G6805-II electro-acupuncture device (Xinsheng Industrial Corporation LTD., Qingdao, China). Prior to the treatment, a 50-hour test was run to assess consistency and calibration of device in measuring known resistors and capacitors. The needles in the points of shenting, baihui, anterior shencong, and posterior Shencong were connected with positive electrode, and the needles in the points of left-sided Shencong, right-sided Shencong, left-sided Fengchi, and right-sided Fengchi were connected with negative electrode, respectively. The two groups were treated with continuous wave of 100 Hz, and the strength of stimulation was tested by the tolerance of patients each time every group. The patients were treated for 30 min each time every other day and continuously treated up to 12 weeks.

### 2.5. Efficacy and Safety Evaluation

At one week before and after treatments, the safety of electro-acupuncture head points on treatment of the 512 Wenchuan earthquake-caused PTSD was evaluated by the measurement of body temperature, blood pressure, pulse, respiration, blood parameters, liver and kidney function, and so forth. The efficacy and side effects of treatment methods were also evaluated using Clinical-Administered PTSD Scale (CAPS), Hamilton Depression Scale (HAMD), Hamilton Anxiety Scale (HAMA), and Treatment Emergent Symptom Scale (TESS) according to clinical data. The three-scale scores of CAPS, HAMD, and HAMA were analyzed and calculated the integral reduced rate at before treatment, after 6 weeks of treatment, and after 12 weeks of treatment. Moreover, results of CAPS were also recorded and calculated the integral reduced rate by followup at 3 and 6 months. Reduction rate is equal (last  score − this  score)/last score. The scale score of TESS was recorded for evaluating the side effects of treatment method during the treatment period. 

### 2.6. Safety Evaluation Standard

In the present study, the safety evaluation standards were divided into four levels, (1) those that no any adverse reaction was observed; (2) those that have a little adverse reactions, but the treatment may continue without any process; (3) those that moderate adverse reactions were observed, and the treatment may continue with some process or help; (4) those that adverse events caused the discontinuation of treatment. 

### 2.7. Statistical Analysis

Clinical experiments were designed by Research Department of Hong Kong and Hang Seng School of Commerce, and data was analyzed using SPSS 16.0 software package. *P* values less than 0.05 were considered statistically significant.

## 3. Results

### 3.1. Baseline Characteristics

Participant characteristics for the 5.12 Wenchuan earthquake-caused SCI patients were shown in Table 1. Table 1 showed that there were no reliable differences between assigned groups by sex number and age.

**Figure 2:**
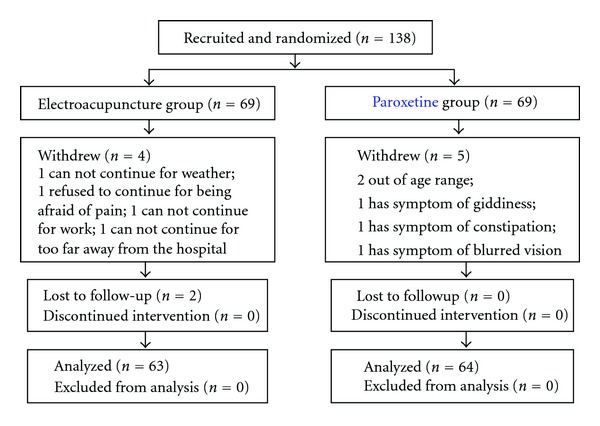
Flow chart of the study sample.

Figure 2 showed the flow of participant randomization, withdrawal, and completion. 138 participants were randomized divided into electro-acupuncture and paroxetine groups, and 69 began the protocol in the two groups. 65 (94.2%) and 64 (92.8%) completed treatment in the electro-acupuncture and medication groups, respectively. Four patients from the electro-acupuncture group were withdrew for weather, pain, work, and too far distance, respectively. In the medication group, two patients dropped out due to age, and three dropped out due an allergic reaction to paroxetine. End treatment and 3- and 6-month followup assessments were obtained in the electro-acupuncture and medication groups for 63 and 64 participants, respectively. Moreover, our results showed that sex, age, and compliance as well as the score of pretreatment CAPS, HAMD, and HAMA in two groups are not statistically significant.

### 3.2. Scores in CAPS, HAMA, HAMD before and after Treatment

As shown in Table 2, comparison of scores in CAPS suggested that the scores in the two groups are not significant differences before and after treatment (*P* > 0.05). Similarly, the scores in HAMA and HAMD also showed no significant differences between the intergroup before and after treatment (*P* > 0.05). 

### 3.3. Reduction Rate in CAPS, HAMA, HAMD before and after Treatment

As shown in Table 3, the reduced rate of CAPS in the treatment group was significantly higher than those in the paroxetine group after treatment and followup and has significant difference between the intergroup (*P* < 0.05). Comparing reduction rate in the control groups, there were significant differences compared to those before treatment (*P* < 0.05). In the treated group, the reduced rates of HAMD are 39.8 ± 26.1, 59 ± 28.1, 70.7 ± 21.7 and 77.1 ± 20.1 at 6 weeks treatment, 12 weeks treatment, 3-months followup and 6-month followup, respectively. These results showed that the treatment group is better than those in the control groups and showed significant difference between the two groups after treatment (*P* < 0.05). Compared to the paroxetine group, the reduction rates of HAMA increased 35.5%, 24.5%, 32.3%, and 21.5% at 6-week treatment, 12-week treatment, 3-month followup and 6-month followup, respectively. There were significant differences when comparing the reduction rates of HAMA between the intergroup and in the intragroup, respectively (*P* < 0.05).

### 3.4. Safety Evaluation

As shown in the clinical results of TESS, 185 adverse events on behavior, autonomic nerve, cardiovascular system, and so forth, were observed in the paroxetine group. The side effects on behavior were mainly as following: excitement or agitation (4 patients, 2.16%), depression (3 patients, 1.62%), activity increased (2 patients, 1.08%), activity declined (6 patients, 3.24%), insomnia (28 patients, 15.1%), and fatigue (7 patients, 3.78%). The side effects autonomic nerve mainly included xerophthalmia (36 patients, 3.78%), stuffy nose (1 patients, 0.54%), blurred vision (3 patients, 1.62%), constipation (17 patients, 9.19%), saliva increase (8 patients, 4.32%), sweat (11 patients, 5.95%), nausea and vomiting (11 patients, 5.95%), and diarrhea (6 patients, 3.24%). The side effects on cardiovascular system were mainly dizziness (5 patients, 2.70%), tachycardia (3 patients, 2.62%), and skin allergy symptom (1 patients, 0.54%). The side effects of appetite loss/anorexia (21 patients, 11.4%) and headache (11 patients, 5.95%) were observed. A systematic review of prospective studies of acupuncture safety found the most common adverse events were needle pain, hematoma, faint during acupuncture treatment, and bleeding. In the electro-acupuncture group, the most frequent side effects reported by the patients were minor needle pain (24 patients, 39.3%), minor superficial bleeding (27 patients, 44.3%), and minor hematoma (9 patients, 14.8%) which were experienced during acupuncture. Only one case of moderate pain (1.64%) was reported in 61 adverse events. Moreover, results from the body temperature, blood parameters, pulse, and respiration of all patients, as well as liver and kidney function showed that no significant changes are observed in the electro-acupuncture treatment. However, the leukocyte count of one patient in the control group decreased significantly, which is related to the side effects of paroxetine. The patient stopped taking paroxetine and was given appropriate treatment. After two weeks, the leukocytes reached normal level. These findings suggested that the efficacy and safety of electro-acupuncture method on PTSD treatment are relatively better than those of the paroxetine method.

## 4. Discussion

In the view of Western medicine, PTSD is primarily caused by a combination of rauma mental troubles and mental disorders in disaster, as well as interference with the normal emotions and work of the neurosis. PTSD can continue for years, and its symptoms can affect every life domain physiologically, psychologically, occupationally, and socially [6]. Thus, there are ongoing clinical guidelines about the optimal diagnosis and treatment of PTSD, and the relevant research literature has burgeoned in recent decades. Due to the long-lasting and resistant symptoms, the treatment of PTSD is complex, both in terms of available treatments and the myriad of trauma possibilities that cause it. Treatment for the symptoms of PTSD involves three approaches either alone or in combination: psychopharmacology, psychotherapy, and education and supportive measures. Pharmacotherapy proved to be partially effective in reduction of symptoms of increased irritability and reexperiencing, but most often it had no effect on symptoms of avoidance. Along with novel agents came to be used for drug treatment, but the results were only partially satisfactory [19]. To date, no specific pharmacological interventions can be recommended as efficacious in preventing the development of PTSD in at-risk individuals. Moreover, many patients are resistant to the idea of pharmaceutical therapies for their disease due to the side effect profiles and adverse reactions for the drugs typically prescribed as part of their treatment regimens. Thus, patients are forced to choose between suffering with PTSD or suffering with the side effects of the medications used to treat it. Given the barriers and drawbacks associated with traditional interventions, acupuncture is emerging as a promising complementary and alternative treatment for PTSD.

Acupuncture is accepted as an effective treatment for many symptoms and disorders, including neuropsychiatric disorders. The number of patients choosing acupuncture as an alternative treatment is increasingly parallel to growing scientific evidence supporting the efficacy of acupuncture [12]. No undesirable side effect has been observed and it is thought that EA may be an alternative to other methods of pain relief during labor or alleviate cerebral ischemia damage [18]. In electro-acupuncture stimulators are used to give pulsatile stimulation parting passive contractions to muscles/group of muscles. Electro-acupuncture achieves early results in certain conditions and is specifically indicated in treatment of palsies and in pain management [20]. Moreover, EA has been shown to alter polycystic ovaries induced by steroids through regulation of ovarian nerve growth factors [21]. In both animals and humans the response in various CNS targets is dependent on the type of acupuncture and the frequency of stimulation in the case of electro-acupuncture [22].

In Traditional Chinese Medicine, the symptom of PTSD belongs to scope of “emotional diseases” or “Depression,” if diseases occur in the head, Baihui (GV 20), Sishencong (Ex-HN 1), Shenting (GV 24), and Fengchi (GB 20), and so forth, which are closer to the precentral gyrus [23, 24]. Earlier reports suggested that a popular acupoint for mental and nervous system diseases is Baihui; additional acupoints include Sishencong (Ex-HN 1), which would enhance the efficacy of Baihui [25]. The reason for Baihui (GV 20) needling is predominantly to treat psychological maladies, as there are general sedative and harmonizing effects. This point is ordinarily used in every acupuncture treatment because of its general psychological effects; it may also be effective for treating insomnia, anxiety, headaches, apoplexy, and weakness of memory. The frontal lobe is mainly responsible for these disorders or symptoms [26]. The mechanism for acupuncture's potential effect on Attention Deficit Disorder (ADHD) is still being researched; however, the acupoints chosen as suitable for the treatment of ADHD are the Shenting (GV 24) and Sishencong (EX-HN 1) [27]. Studies indicated that the Fengchi point has seven general functions of dispelling pathogenic wind, purging pathogenic fire, relieving the depressed liver-qi, resolving the phlegm, activating the blood, tranquilizing the mind, and checking spasm and convulsion [28]. 

In the present study, our results suggested that the scores of CAPS, HAMA, and HAMD in the two groups show no significant differences before and after treatment (*P* > 0.05). Moreover, the reduced rate of CAPS, HAMA, and HAMD in the treatment group was significantly higher than those in the control group after treatment and followup and have significant difference in the intergroup (*P* < 0.05). Clinical studies on poststroke depression (PSD) patients suggested that acupuncture is effective in improving poststroke depression in PSD patients when Sishencong (EX-HN 1), Baihui (GV 20), Shenting (GV 24), were used as the major acupoints [29]. Depression patients were treated with electro-acupuncture, by selecting Baihui (GV 20), Shenting (GV 24), or Sishencong (Ex-HN 1) (in alternation) as the major acupoints, and the results showed the depressive symptoms were obviously relieved in the treatment group, and the survival quality of the patients was elevated at the same time, remarkably better than the control group [30]. Based on the above results, our findings provide preliminary support for the use of Baihui (GV 20), Sishencong (EX-HN 1), Shenting (GV 24), and Fengchi (GB 20) by electro-acupuncture therapy in treating earthquake survivors with PTSD. Moreover, results of side effects showed that the incidence of adverse effects is substantially lower with electro-acupuncture than with paroxetine treatment. Thus, the present electro-acupuncture method may provide an alternative therapy that is safe, effective, and reasonably cheap for PTSD patients.

## 5. Conclusion

The efficacy and safety of electro-acupuncture at selected acupoints have been investigated to treatment 512 Wenchuan earthquake-caused PTSD patients using Randomized Controlled Trials (RCTs). The present study suggested that both electro-acupuncture and medication therapy may improve the symptoms of PTSD patients; the former is better than the latter in efficacy. Although acupuncture was shown to be of value as a therapeutic intervention for PTSD patients, further definitive research about simultaneous clinical and biological effects is needed to support the use of electro-acupuncture for PTSD treatment.

## Figures and Tables

**Table 1:** Participant characteristics of the two groups.

Characteristic	Paroxetine	Electroacupuncture	*P* value
Sex number (%) men	28 (43.7)	26 (40)	0.778
Sex number (%) women	36 (56.3)	39 (60)	
Age, mean (SD)	50.3 ± 12.3	48.3 ± 13.3	0.562

**Table 2:** Comparison of scores in CAPS, HAMA, and HAMD before and after treatment.

Score	Time/group	Before	6 weeks	12 weeks	3-month followup	6-month followup	*P* and *F* ^b^
CAPS	Paroxetine (*n* = 64)	66.8 ± 21.3	44.7 ± 22.4	31.2 ± 18.9	26.3 ± 19.4	21.8 ± 18.7	*P* = 0.000
	Treatment (*n* = 63)	65.8 ± 19.7	39 ± 20.3	27.3 ± 17.9	20.4 ± 15.5	15.9 ± 14.3	*F* = 453.4
	*P* and *F* ^a^	*P* = 0.132	*F* = 2.30				

HAMD	Paroxetine (*n* = 64)	12.7 ± 5.2	8.38 ± 4.28	6.11 ± 3.89	4.88 ± 3.65	4.25 ± 3.62	*P* = 0.00
	Treatment (*n* = 63)	13.1 ± 5.56	7.69 ± 4.44	5.22 ± 3.86	3.69 ± 3.06	2.98 ± 2.99	*F* = 332.3
	*P* and *F* ^a^	*P* = 0.255	*F* = 1.31				

HAMA	Paroxetine (*n* = 64)	11.7 ± 5.85	7.61 ± 3.86	5.59 ± 3.18	4.70 ± 3.2	3.86 ± 3.15	*P* = 0.00
	Treatment (*n* = 63)	11.6 ± 5.11	6.38 ± 3.53	4.65 ± 3.44	3.46 ± 2.94	2.95 ± 2.85	*F* = 204.9
	*P* and *F* ^a^	*P* = 0.101	*F* = 2.74				

Note: ^a^for the intergroup comparison; ^b^for the intragroup comparison.

**Table 3:** Comparison of reduction rate in CAPS, HAMA, and HAMD before and after treatment.

Score	Time/group	6 weeks	12 weeks	3-month followup	6-month followup	*P* and *F* ^b^
CAPS	Control (*n* = 64)	33.3 ± 25.5	54 ± 21.2	61.7 ± 22.1	68.6 ± 22.4	*P* = 0.00
	Treatment (*n* = 63)	41.9 ± 22.2	60.3 ± 20.8	70.4 ± 18.3	77.2 ± 17.1	*F* = 258.6
	*P* and *F* ^a^	*P* = 0.019	*F* = 5.64			

HAMD	Control (*n* = 64)	31.4 ± 25.9	49.4 ± 27.9	59.6 ± 28.7	64.1 ± 31.8	*P* = 0.00
	Treatment (*n* = 63)	39.8 ± 26.1	59 ± 28.1	70.7 ± 21.7	77.1 ± 20.1	*F* = 181.5
	*P* and *F* ^a^	*P* = 0.014	*F* = 6.20			

HAMA	Control (*n* = 64)	29.9 ± 25	46.1 ± 29.8	52 ± 36.2	60.9 ± 32.9	*P* = 0.000
	Treatment (*n* = 63)	40.5 ± 25.5	57.4 ± 26.7	68.8 ± 21.7	74 ± 20.4	*F* = 122
	*P* and *F* ^a^	*P* = 0.004	*F* = 8.82			

Note: ^a^the intergroup comparison; ^b^the intragroup comparison.

## References

[1] Feldman DB (2011). Posttraumatic stress disorder at the end of life: extant research and proposed psychosocial treatment approach. *Palliative & Supportive Care*.

[2] Player MS, Peterson LE (2011). Anxiety disorders, hypertension, and cardiovascular risk: a review. *International Journal of Psychiatry in Medicine*.

[3] Bisson J, Andrew M (2007). Psychological treatment of post-traumatic stress disorder (PTSD). *Cochrane Database of Systematic Reviews*.

[4] Pratchett LC, Daly K, Bierer LM (2011). New approaches to combining pharmacotherapy and psychotherapy for posttraumatic stress disorder. *Expert Opinion on Pharmacotherapy*.

[5] Frueh BC, Grubaugh AL, Cusack KJ, Kimble MO, Elhai JD, Knapp RG (2009). Exposure-based cognitive-behavioral treatment of PTSD in adults with schizophrenia or schizoaffective disorder: a pilot study. *Journal of Anxiety Disorders*.

[6] Ravindran LN, Stein MB (2009). Pharmacotherapy of PTSD: premises, principles, and priorities. *Brain Research*.

[7] Benedek DM, Friedman MJ, Zatzick D Guideline Watch (March 2009), Practice Guideline for the Treatment of Patients with Acute Stress Disorder and Posttraumatic Stress Disorder.

[8] Earthquake Bureau of Sichuan Province Report about quake hit cities and counties of Wenchuan earthquake on May 12, 8.0 on the Richter scale, affected by tectonic activity.

[9] Zhao GQ, Wang YG, Wang YQ (2008). Prevalence and predictors of acute stress disorder after earthquake: findings from Wenchuan earthquake in China. *Zhonghua Yu Fang Yi Xue Za Zhi*.

[10] Wang XL, Tao J, Li LJ (2009). Screening for post-traumatic stress disorder symptoms and its influence factors among victims in temporary settlements early after Wenchuan Earthquake. *China Medicine*.

[11] Fan F, Zhang Y, Yang Y, Mo L, Liu X (2011). Symptoms of posttraumatic stress disorder, depression, and anxiety among adolescents following the 2008 Wenchuan earthquake in China. *Journal of Traumatic Stress*.

[12] Hollifield M (2011). Acupuncture for posttraumatic stress disorder: conceptual, clinical, and biological data support further research. *CNS Neuroscience & Therapeutics*.

[13] Hollifield M, Sinclair-Lian N, Warner TD, Hammerschlag R (2007). Acupuncture for posttraumatic stress disorder: a randomized controlled pilot trial. *Journal of Nervous and Mental Disease*.

[14] Smith CA, Hay PPJ, Macpherson H (2010). Acupuncture for depression. *Cochrane Database of Systematic Reviews*.

[15] Feinstein D (2010). Rapid treatment of ptsd: why psychological exposure with acupoint tapping may be effective. *Psychotherapy*.

[16] Spence DW, Kayumov L, Chen A (2004). Acupuncture increases nocturnal melatonin secretion and reduces insomnia and anxiety: a preliminary report. *Journal of Neuropsychiatry & Clinical Neurosciences*.

[17] Han JS (1986). Electroacupuncture: an alternative to antidepressants for treating affective diseases?. *The International Journal of Neuroscience*.

[18] Chen WX, Wu-Li L, Wong VCN (2008). Electroacupuncture for children with autism spectrum disorder: pilot study of 2 cases. *Journal of Alternative and Complementary Medicine*.

[19] Alderman CP, McCarthy LC, Marwood AC (2009). Pharmacotherapy for post-traumatic stress disorder. *Expert Review of Clinical Pharmacology*.

[20] Bokhari SZH, Zahid S (2007). Pain management in lumbago: role of acupuncture in addition to local steroid infiltration at trigger points. *Journal of Postgraduate Medical Institute*.

[21] Mani L, Roco ML, Barbaro Paparo S, Guaragna M (2011). Electroacupucture and nerve growth factor: potential clinical applications. *Archives Italiennes de Biologie*.

[22] Napadow V, Makris N, Liu J, Kettner NW, Kwong KK, Hui KKS (2005). Effects of electroacupuncture versus manual acupuncture on the human brain as measured by fMRI. *Human Brain Mapping*.

[23] Cao X, Li WZ, Ming L (2005). Progress of study on relevant influential factors and mechanism of post-stroke depression. *An Hui Yi Yao*.

[24] Chen XY, Zhu Y, Huang XS (2009). Effect of strong stimulation of acupuncture at twelve Jing-well points as main for neurosurgery patients with disorder of consciousness. *Zhongguo Zhen Jiu*.

[25] Zhang E (1990). *Chinese Acupuncture and Moxibustion in a Practical English-Chinese Library of TCM*.

[26] Han C, Li X, Luo H, Zhao X, Li X (2004). Clinical study on electro-acupuncture treatment for 30 cases of mental depression. *Journal of Traditional Chinese Medicine*.

[27] Li S, Yu B, Zhou D (2011). Acupuncture for attention deficit hyperactivity disorder (ADHD) in children and adolescents. *Cochrane Database of Systematic Reviews*.

[28] Shanand BZ, Shao SJ (1999). Clinical application of Fengchi (GB 20). *World Journal of Acupuncture-Moxibustion*.

[29] Wu JP (2010). Clinical observation on acupuncture treatment of 150 cases of post-stroke depression according to syndrome differentiation. *Zhen Ci Yan Jiu*.

[30] Tang JX, Guan NH, Li L (2003). Influence of electroacupuncture on life quality in patients with post-apoplectic depression. *Shang Hai Zhen Jiu Za Zhi*.

